# Use of Intermittent Preventive Treatment among Pregnant Women in Sub-Saharan Africa: Evidence from Malaria Indicator Surveys

**DOI:** 10.3390/tropicalmed3010018

**Published:** 2018-02-11

**Authors:** Sanni Yaya, Olalekan A. Uthman, Agbessi Amouzou, Ghose Bishwajit

**Affiliations:** 1 School of International Development and Global Studies, University of Ottawa, Ottawa, ON K1N 6N5, Canada; sanni.yaya@uottawa.ca; 2 Warwick Centre for Applied Health Research and Delivery (WCAHRD), Division of Health Sciences, Warwick Medical School, University of Warwick, Coventry CV4 7AL, UK; olalekan.uthman@warwick.ac.uk; 3 Bloomberg School of Public Health, Johns Hopkins University, Baltimore, MD 21205, USA; aamouzo1@jhu.edu

**Keywords:** malaria in pregnancy, Malaria Indicator Survey, IPTp, sub-Saharan Africa

## Abstract

Uptake of intermittent preventive therapy in pregnancy (IPTp) with sulfadoxine-pyrimethamine (IPTp-SP) is a clinically-proven method to prevent the adverse outcomes of malaria in pregnancy (MiP) for the mother, her foetus, and the neonates. The majority of countries in sub-Saharan Africa have introduced IPTp policies for pregnant women during the past decade. Nonetheless, progress towards improving IPTp coverage remains dismal, with widespread regional and socioeconomic disparities in the utilisation of this highly cost-effective service. In the present study, our main objective was to measure the prevalence of IPTp uptake in selected malaria-endemic countries in sub-Saharan Africa, and to investigate the patterns of IPTp uptake among different educational and wealth categories adjusted for relevant sociodemographic factors. For this study, cross-sectional data on 18,603 women aged between 15 and 49 years were collected from the Malaria Indicator Surveys (MIS) conducted in Burkina Faso, Ghana, Mali, Malawi, Kenya, Nigeria, Sierra Leone, and Uganda. The outcome variable was taking three doses of IPTp-SP in the last pregnancy, defined as adequate by the WHO. According to the analysis, the overall prevalence of taking three doses of IPTp-SP in the latest pregnancy was 29.5% (95% CI = 28.2–30.5), with the prevalence being highest for Ghana (60%, 95% CI = 57.1–62.8), followed by Kenya (37%, 95% CI = 35.3–39.2) and Sierra Leone (31%, 95% CI = 29.2–33.4). Women from non-poor households (richer—20.7%, middle—21.2%, richest—18.1%) had a slightly higher proportion of taking three doses of IPTp-SP compared with those from poorest (19.0%) and poorer (21.1%) households. Regression analysis revealed an inverse association between uptake of IPTp-SP and educational level. With regard to wealth status, compared with women living in the richest households, those in the poorest, poorer, middle, and richer households had significantly higher odds of not taking at least three doses of IPTp-SP during their last pregnancy. The present study concludes that the prevalence of IPTp-SP is still alarmingly low and is significantly associated with individual education and household wealth gradient. Apart from the key finding of socioeconomic disparities within countries, were the between-country variations that should be regarded as a marker of inadequate policy and healthcare system performance in the respective countries. More in-depth and longitudinal studies are required to understand the barriers to, and preferences of, using IPTp-SP among women from different socioeconomic backgrounds.

## 1. Introduction

Malaria is historically recognised as a significant public health concern for healthcare systems in sub-Saharan Africa (SSA), and to date remains a major contributor to maternal and infant morbidity and mortality in the continent [1,2,3]. Led by strong political commitments and programmatic efforts by national and international agencies, most countries in SSA have achieved noteworthy progress in increasing the coverage of insecticide-treated nets (ITNs) and antimalarial intermittent preventive therapy in pregnancy (IPTp) that has brought about a subsequent reduction in the prevalence of malaria in the population [4,5,6]. There is a growing body of evidence that in the endemic regions malaria in pregnancy (MiP) remains a prominent preventable cause of maternal and infant morbidity and mortality [7], accounting for about 75,000–200,000 infant deaths in the region [8,9]. Despite this evidence and the ongoing malaria elimination efforts, the rate of utilisation of preventive measures, especially IPTp, is still unsatisfactory and is far from being universal [4].

Commonly known as a disease of poverty, there are also certain demographic patterns in the distribution of the epidemic that cause pregnant women and infants to bear the greatest risk of the most severe clinical symptoms of malaria [10]. MiP is associated with a range of complications for mothers, fetuses, and neonates including maternal hypoglycaemia, intrauterine growth retardation, placental malaria (PM), fetal hypotrophy, miscarriage, preterm delivery, and low birth weight, which explains the contribution of malaria to higher maternal and neonatal mortality rates [11,12,13]. The majority of the complications of MiP are caused by *Plasmodium falciparum*, although the severity of the symptoms and complications can vary across regions and the degree of acquired immunity among the individuals. Mechanisms responsible for heightened susceptibility to malaria during gestational period have been explained by clinical studies. In *P. falciparum* infection, the infected erythrocytes are reported to sequester in the placenta and produce a unique type of surface antigen (unique variant surface antigens (VSA) [14] that is more likely to go unrecognized due to a lower immune system response during pregnancy [15]. In the case of primigravid woman, the risk of severity can be ever greater because there is no acquired immunity for malaria, especially if the diagnosis is made late or if *P. falciparum* shows resistance to antimalarial drugs or malaria chemoprophylaxis [16]. Owing to the increased susceptibility to infection during gestation, pregnant women are more likely to develop severe disease than their non-pregnant counterparts and hence are encouraged to take antimalarial IPTp [2].

Although about 37 countries has so far introduced IPTp policy [17], poor implementation and enforcement of these policies are reflected through low coverage and disparities in utilisation rates [5,18,19,20,21]. According to a review study conducted in 2009, the rate of antimalarial drugs uptake among pregnant women varied from as low as 2% to more than 60% with significant regional and socioeconomic disparities in utilisation rates [22]. Apart from the healthcare and financial barriers, previous studies have reported various individual and community-related barriers to the utilisation of IPTp-SP, including delayed initiation of antenatal care attendance, poor awareness and knowledge about MiP and the preventive measures [23], stock-outs of free of cost IPTp-SP [20], and spousal discord [24]. In the wake of this situation, the American Society of Tropical Medicine and Hygiene held a global ‘Call to Action’ seminar in 2014 with representatives from several countries in SSA to discuss opportunities for scaling up the coverage of IPTp [4]. As more and more countries in Asia and South America are approaching malaria elimination and global donor funding for malaria programs are becoming increasingly scarce [25], countries in SSA are faced with the challenge of continuing the fight against malaria and addressing the persisting barrier to adoption of preventive measures in the population. Achieving the target of universal coverage for IPTp is undoubtedly a huge task, one that needs to be leveraged by more innovative and pro-poor strategies by reducing the accessibility and affordability barriers to utilisation for which evidence from population-based studies is absolutely crucial. In this regard, we undertook the present study based on secondary data from Malaria Indicator Surveys (MIS) conducted in selected malaria-endemic countries in SSA. The main objectives were to provide updated information regarding the prevalence of IPTp utilization and to investigate the influence of any socioeconomic factors in the utilization status.

## 2. Methods

### 2.1. Countries Included in the Study

**Figure 1:**
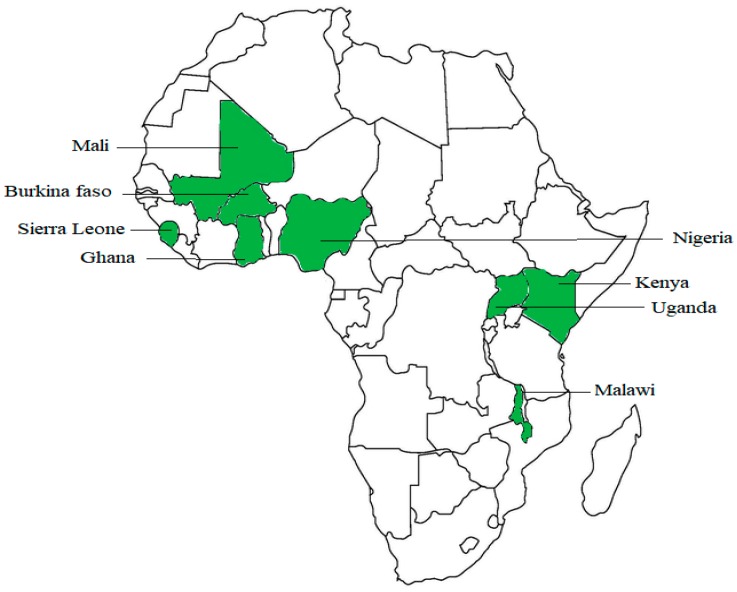
Countries included in the study.

Data were obtained from the Malaria Indicator Surveys (MIS) conducted in eight sub-Saharan African countries as shown in Figure 1: Burkina Faso (2014), Ghana (2016), Mali (2015), Malawi (2014), Kenya (2015), Nigeria (2015), Sierra Leone (2016), and Uganda (2014–2015). Surveys were conducted by technical assistance as well as funding by ICF International through the Demographic and Health Survey (DHS) Program, which is a USAID-funded project providing support and technical assistance in the implementation of population and health surveys in countries worldwide [21].

The respective institutions implementing the surveys are as follows. Burkina Faso: Institut National de la Statistique et de la Démographie (INSD); Ghana: Ghana Statistical Service (GSS), Kenya: National Malaria Control Programme (NMCP) of the Ministry of Health and the Kenya National Bureau of Statistics (KNBS); Malawi: National Malaria Control Programme (NMCP) of the Ministry of Health; Mali: INFO-STAT en collaboration avec le Programme National de Lutte contre le Paludisme (PNLP), Ministère de la Santé et de l’Hygiène Publique, et l’Institut National de la Statistique (INSTAT), Ministère de l’Aménagement du Territoire et de la Population; Sierra Leone: National Malaria Control Programme (NMCP), Statistics Sierra Leone (SSL), the College of Medicine and Allied Health Services (COMAHS) of the University of Sierra Leone (USL), and Catholic Relief Services (CRS); Nigeria: National Malaria Elimination Programme (NMEP), the National Population Commission (NPopC), and the National Bureau of Statistics (NBS); and Uganda: Uganda Bureau of Statistics (UBOS) and the National Malaria Control Programme (NMCP) of the Uganda Ministry of Health.

### 2.2. Survey Design and Objectives

MIS surveys employ stratified two-stage cluster design. In the first stage, sampling strata were created, from which clusters are selected per stratum by a probability-proportional-to-size selection. A complete listing in the selected clusters serves as the sampling frame for the second stage. In the second stage, households are selected from each cluster by equal probability systematic sampling. Study participants were women aged 15–49 years residing in non-institutional places in urban and rural areas.

The objectives of the surveys are to provide quality data for measuring progress toward selected targets and goals required for effective monitoring, and assessing the national malaria program’s implementation and measurement. Specifically, the surveys aim to measure the indicators of ownership and use of mosquito bed nets; coverage of the intermittent preventive treatment programme for pregnant women; treatment-seeking behaviour; measure indicators of knowledge, attitudes, and behaviour related to malaria control; and to determine the factors associated with malaria and anaemia. Besides these questions, many other questions about basic demographics, education, use of ITNs, malaria prevention practices during pregnancy, and knowledge of malaria are surveyed. Further details on survey are available from the data sources [26].

### 2.3. Variables

Outcome variable: The main outcome variable was adequate use of intermittent preventive therapy with sulfadoxine-pyrimethamine (IPTp-SP). It was assessed by asking the respondents whether or not they took Fansidar (sulfadoxine-pyrimethamine, SP) during their last pregnancy, and how many times it was taken. Administration of IPTp-SP during antenatal visits is considered to be one of the three key interventions recommended by the World Health Organization (WHO) for controlling MiP in stable malaria transmission areas [4]. Prophylactic administration of SP has been found to be effective in preventing the consequences of MiP (e.g., low birthweight) [27]. As WHO recommends at least three doses of SP, for the present study we used the recommendation of three doses to define the adequacy of IPTp-SP dosage: adequate (>2 doses) and inadequate (≤2 doses).

Independent variables were age: 15–19/20–24/25–29/30–34/35–39/40–44/45–49; setting: urban/rural; education: no education/primary/secondary/higher; religion: Islam/Christian/Other; wealth index: poorest/poorer/middle/richer/richest; received malaria information on TV: no/yes; received malaria information on radio: no/yes; received malaria information from health workers: no/yes.

All the variables were self-reported, except for the wealth index. For the calculation of household wealth status, instead of direct income, the volume of durable goods (e.g., TV, radio, and bicycle) possessed by the household as well as and housing quality (e.g., type of floor, wall, and roof) are taken into consideration. Each item is assigned a factor score generated through principal component analysis (PCA), which is then summed and standardized for the households. These standardized scores place the households in a continuous scale based on relative wealth scores. The scores are thus obtained from a continuous scale and subsequently categorized into quintiles to rank the household as poorest/poorer/middle/richer/richest to richest [28]. For the present study, households in lowest two categories were merged and categorized as poor, and those from middle to richest were merged as non-poor.

### 2.4. Data Analysis

Statistical analyses were performed with SPSS version 24. Firstly, the datasets were cleaned for outliers, checked for potential multicollinearity, and then merged to perform pooled analysis. Due to nonproportional allocation of the samples across the survey regions, they were not self-weighting. Furthermore, MIS surveys use cluster sampling design. To adjust for sample weight and cluster sampling techniques, the analysis was preceded by the preparation of complex survey file by accounting for primary sampling units, sample strata, and sample weight. Prevalence rates of adequate uptake of IPTp-SP for each explanatory variable were shown as percentages. Country-wise prevalence of adequate uptake was presented as bubble charts with 95% CIs. After that, binary logistic regression model was used to calculate the odds ratios of the associations between adequate of IPTp-SP and women’s educational level and household wealth status while adjusting for potential confounding variables. Results of regression analysis were presented as odds ratios along with their 95% CIs as an indicator of significance, as well as of the precision of the OR values. For all associations, *p*-value of <0.05 was considered statistically significant.

### 2.5. Ethical Approval

The study was based on analysis of anonymized secondary data available in the public domain of DHS; therefore, no additional approval was necessary. However, approval for the reuse of the data was obtained by authors from DHS.

## 3. Results

### 3.1. Descriptive Statistics

Basic demographic and socioeconomic information of the sample population was summarized in Table 1. In total, 18,603 women aged between 15–49 years were included in the present study. Mean age of the participants was 28.76 years (SD 7.01). The overall prevalence of taking adequate doses of IPTp-SP during the latest pregnancy was 29.5% (95% CI = 28.2–30.5). As clarified in the table, this prevalence was higher among women aged between 25–29 years who lived in rural settings, had no education, lived in non-poor households, and received malaria-related information from television, radio, and health workers.

**Figure 2:**
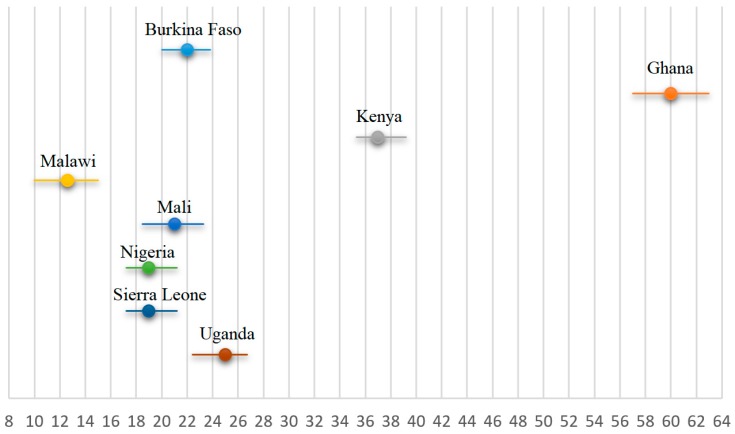
Percentage of women receiving at least three doses of IPTp-SP during last pregnancy in selected sub-Saharan countries.

Figure 2 shows the percentage of women who received three doses of SP during last pregnancy in the individual countries. Among the eight countries, only Ghana had a prevalence of three doses of SP taken for more than half of the women (60%), while in Malawi, Nigeria, and Uganda, more than four-fifth of the women did not get the adequate dosage of SP.

### 3.2. Regression Analysis on the Association between Educational Level and Wealth Status with the Uptake of IPTp-SP

Results of regression analysis was summarized in Table 2. As the results indicate, we found a negative association between the uptake of IPTp-SP with educational status, and positive association with wealth status. Compared with women who had no formal education, the odds of inadequate uptake (<3 doses) of IPTp-SP were higher among those with primary (OR = 1.307, 95% CI = 1.104–1.547), secondary (OR = 1.431, 95% CI = 1.202–1.705), and higher (OR = 1.658, 95% CI = 1.401–1.962) educational status. With regard to wealth status, women living in the poorest, poorer, middle, and richer households had respectively 32% (OR = 1.320, 95% CI = 1.140–1.529), 24% (OR = 1.235, 95% CI = 1.069–1.425), 21% (OR = 1.210, 95% CI = 1.054–1.388), 21.2% (OR = 1.212, 95% CI = 1.069–1.374) higher odds of not taking at least three doses of IPTp-SP during their last pregnancy.

For sensitivity analysis, we ran the regression models by taking two doses of SP as an outcome variable that generated similar results of inverse relationship between the uptake of IPTp-SP with educational status, and positive association with wealth status (results not shown). Model fitness was evaluated by Nagelkerke R squared statistics, which showed a satisfactory model fit, as the fully adjusted model explained 48% of the variance in the outcome variable.

## 4. Discussion and Policy Recommendation

Our findings revealed that the overall prevalence of adequate uptake of IPTp-SP was remarkably low, as less than a quarter of the women reported taking at least three doses of SP during their last pregnancy. This is far below Roll Back Malaria’s (RBM) target of 80% coverage of ≥2 doses of IPTp-SP^20^. Poor coverage and utilisation of IPTp-SP is a serious concern for malaria prevention and eradication programs for health NGOs and national governments in SSA [19,29]. There is mounting evidence regarding the failure of the healthcare systems to promote maternal healthcare services utilisation due to persisting infrastructural, sociocultural, and financial barriers [30,31]. Thus, it makes sense that even if IPTp is distributed free or at minimal cost, a great proportion of the women may remain deprived of antimalarial services due to poor exposure to antenatal contacts. Although we were not able to assess the influence of antenatal care, in light of the past evidence it is arguable that improving antenatal care attendances by addressing the contextual barriers might eventually prove beneficial for malaria prevention programs by increasing the exposure to preventive measures [32,33].

Apart from the low average, there were considerable inter- and intra-country variations in the adequate uptake of IPTp as well. There are certain difficulties in comparing these findings with previous ones due to methodological differences. Nonetheless, inadequate use of SP was reported in Cameroon [34], Gabon [35], Burkina Faso [36], and Benin [37]. A meta-analysis study conducted in 2013 on African countries reported very low prevalence of IPTp uptake; only a quarter of the women received only one dose [17]. Coverage of IPTp appears to be comparatively better in Ghana and Kenya, who were among the earliest countries to approve IPTp policy. The prevalence of taking one dose of IPTp in these two countries was reported to be 80.7% and 35.5%, respectively [29]. In comparison, the corresponding rates were below 10% in Somalia, Benin, and Congo, while, respectively, they were 12% and 11.8% in Madagascar and Central African Republic [38].

For effectively addressing the poor coverage and utilisation rates, it is absolutely essential to identify the limiting factors in order to developing evidence-based intervention strategies. In this study, our choice of predicting the factors was limited, as the data were secondary. However, we assessed the predictability of individual educational level and household wealth status for the uptake of IPTp-SP. The role of individual’s socioeconomic status on self-efficacy, awareness, and preventive behaviour is well-documented. Surprisingly, our findings indicated an inverse relationship between educational level of adequate uptake of IPTp-SP. In contrast, a cross-sectional study among rural women in Kenya reported that women who had formal education had higher likelihood of receiving at least one dosage of IPTp-SP [39]. Similarly, positive association between educational status and IPTp-SP uptake was observed in Malawi and Uganda [31,40]. While this seems counterintuitive from the perspective of health service utilisation, it is hard to explain this irregularity in light of the currently analysis, and hence remains a subject for investigation by future studies. A possible reason could be that women with higher educational status are better off financially and can afford healthier living environments, which makes the use of antimalarial apparently less essential.

As expected, our findings indicate that women living in households relatively poorer than richest ones were less likely to report taking 2+ doses of IPTp-SP. This enabling effect of financial well-being on IPTp-SP uptake is in line with previous findings from Kampala, Uganda, and Kibaha District of Tanzania [41,42]. The financial barrier in this regard may appear irrelevant, as these services are usually provided free of charge by public establishments. However, although the actual costs of taking IPTp-SP seem to insignificant, there might be other hidden or perceived costs associated with it, such as transportation costs or charges for antenatal care. Moreover, even if the products come free of cost, the expenses related to administration, logistics, and staff management can create the need for systematic revenue generation that might discourage poor consumers [43]. This can be especially burdensome for women living in extreme poverty and those who rely on husbands or other household members to cover such fees. Therefore, it is suggested that health policy makers focus on finding innovative ways to incentivize service uptake among the ultra-poor, who are usually most at risk of malaria infection.

From the discussions above, it becomes clear that healthcare systems in sub-Saharan Africa targeting malaria prevention and elimination are faced with enormous challenges to achieve optimum coverage and utilization of IPTp-SP. Apart from health infrastructure-related barriers, health policy makers also need to target the sociocultural, economic, and geographic barriers to maximize the outcomes of the ongoing efforts. As malaria disproportionately affects women and children, this adds to their higher vulnerability to poorer socioeconomic status and translates to low health literacy and treatment-seeking behavior [44]. Therefore, special policy attention is required to safeguard women and children by making gender- and child-sensitive health policies an integral part of malaria prevention programs. It is also necessary to bear in mind that encouraging the uptake of antimalarial drugs needs proper regulation to avoid their incorrect use, especially ones that are no longer recommended and can undermine malaria control efforts by fuelling drug resistance [45]. Another significant barrier to making measurable steps towards malaria elimination is the scarcity of quality data. As Richard Cibulskis (co-ordinator of the global malaria programme of WHO) reported, over 90% of the world’s malaria cases are likely to go unreported, and the data systems are weakest in the places where malaria is the most common, and for some African nations there are no good data at all [46]. To ensure long-term and targeted intervention, national governments in sub-Saharan Africa also need to place stress on improving health informatics systems for malaria, developing better data collection and distribution tools, and correctly managing health records.

### Strengths and Limitations

Apart from the contribution to the current literature, this study has some important strengths and limitations that need to be mentioned. The sample size was large and the findings are generalizable for women aged 15–49 years in the studies countries. Among the limitations were the secondary nature of the sample that prevented us from having any control over the measurement and selection of the variables. We were also unable to assess the specific causes behind not using IPTp-SP in the sample population, except for educational and financial status, which are two well-known enabling factors of positive health behavior among both men and women. Neither was it possible for us to identify the causes behind the inverse association between educational status and uptake of IPTp-SP. The prevalence is also subject to recall error, as women had to remember the use of the service during their most recent pregnancy, within the last five years. Last but not least, as the data were cross-sectional, the association cannot guarantee any causation or directionality.

## 5. Conclusions

In conclusion, the prevalence of IPTp-SP was alarmingly low with noticeable intra- and inter-county variations. Despite several important limitations, the findings of the study provide a fresh insight into the current status of IPTp-SP use during pregnancy in the malaria-endemic regions in sub-Saharan Africa. The shockingly low prevalence of IPTp-SP utilization implies the need to strengthen the policy approaches and programmatic actions to address the structural and socioeconomic factors responsible for the substandard performance of the IPTp policies. Further studies are recommended to identify the perceived barriers to taking IPTp-SP among pregnant women in these countries.

## Figures and Tables

**Table 1:** Distribution of sample population across the explanatory variables, MIS 2014–2016.

	Received at Least 3 Doses of IPTp-SP in Last Pregnancy					*p*-Value
	*N* = 18,603	%	%	95% CI Lower	95% CI Upper	
**Age Group (Mean = 28.76)**						0.061
15–19	1372	7.4	7.0	6.2	7.8	
20–24	4184	22.5	22.1	20.8	23.5	
25–29	4829	26.0	25.7	24.4	27.2	
30–34	3919	21.1	21.3	20.0	22.7	
35–39	2697	14.5	14.5	13.5	15.7	
40–44	1208	6.5	7.0	6.3	7.8	
45–49	394	2.1	2.3	1.9	2.9	
**Setting**						<0.001
Urban	5271	28.3	30.6	27.6	33.7	
Rural	13,332	71.7	69.4	66.3	72.4	
**Religion**						<0.001
Islam	8964	48.2	43.8	40.9	46.8	
Christian	6082	32.7	32.0	29.6	34.5	
Other	3557	19.1	24.2	21.3	27.3	
**Education**						<0.001
No education	8960	48.2	44.3	41.9	46.8	
Primary	3895	20.9	22.4	20.9	24.1	
Secondary	3676	19.8	25.4	23.5	27.4	
Higher	2072	11.1	7.8	6.5	9.4	
**Wealth index**						<0.001
Poorest	4071	21.9	19.0	17.4	20.8	
Poorer	3910	21.0	21.1	19.4	23.0	
Middle	3850	20.7	20.7	19.1	22.3	
Richer	3683	19.8	21.2	19.3	23.1	
Richest	3089	16.6	18.1	15.9	20.4	
TV *						0.05
No	15,013	80.7	22.4	20.3	24.7	
Yes	3590	19.3	77.6	75.3	79.7	
Radio *						<0.001
No	10,008	53.8	45.6	43.3	48.0	
Yes	8595	46.2	54.4	52.0	56.7	
Health worker *						<0.001
No	11,515	61.90	34.7	32.1	37.4	
Yes	7088	38.11	65.3	62.6	67.9	

MIS = Malaria Indicator Survey; CI = confidence interval; *p*-values calculated from Chi-square tests of independence; * = refers to receiving malaria-related information from these sources.

**Table 2:** Association between educational level and wealth status with inadequate uptake of IPTp-SP in pregnancy in selected countries in sub-Saharan Africa, MIS 2014–2016.

	Model 1			Model 2			Model 3		
	OR	95% CI Lower	95% CI Upper	OR	95% CI Lower	95% CI Upper	OR	95% CI Lower	95% CI Upper
**Education (Nil)**									
Primary	1.791	1.590	2.018				1.307	1.104	1.547
Secondary	2.043	1.796	2.324				1.431	1.202	1.705
Higher	2.374	2.086	2.701				1.658	1.401	1.962
**Wealth index (Richest)**									
Poorest				1.185	1.069	1.314	1.320	1.140	1.529
Poorer				1.137	1.025	1.262	1.235	1.069	1.425
Middle				1.085	0.977	1.205	1.210	1.054	1.388
Richer				1.127	1.014	1.253	1.212	1.069	1.374
**Nagelkerke R Square**	0.179			0.134			0.484		

OR = odds ratio, CI = confidence interval. *p* < 0.05. Model 1 = only educational status was entered; Model 2 = only wealth index was entered; Model 3 = adjusted for respondents age, setting, religion, receiving malaria information from TV, radio, and health worker.
